# A review of reporting of participant recruitment and retention in RCTs in six major journals

**DOI:** 10.1186/1745-6215-10-52

**Published:** 2009-07-10

**Authors:** Merran Toerien, Sara T Brookes, Chris Metcalfe, Isabel de Salis, Zelda Tomlin, Tim J Peters, Jonathan Sterne, Jenny L Donovan

**Affiliations:** 1 Department of Sociology, The University of York, Heslington, York, YO10 4PS, UK; 2 Department of Social Medicine, University of Bristol, Canynge Hall, 39 Whatley Road, Bristol, BS8 2PS, UK; 3 Department of Primary Care & Public Health, Cardiff University, Heath Park, Cardiff, CF14 4XN, UK; 4 Academic Unit of Primary Health Care, Department of Community Based Medicine, University of Bristol, 25 Belgrave Road, Bristol, BS8 2AA, UK; 5 Previous affiliation: MRC Health Services Research Collaboration

## Abstract

**Background:**

Poor recruitment and retention of participants in randomised controlled trials (RCTs) is problematic but common. Clear and detailed reporting of participant flow is essential to assess the generalisability and comparability of RCTs. Despite improved reporting since the implementation of the CONSORT statement, important problems remain. This paper aims: (i) to update and extend previous reviews evaluating reporting of participant recruitment and retention in RCTs; (ii) to quantify the level of participation throughout RCTs.

**Methods:**

We reviewed all reports of RCTs of health care interventions and/or processes with individual randomisation, published July–December 2004 in six major journals. Short, secondary or interim reports, and Phase I/II trials were excluded. Data recorded were: general RCT details; inclusion of flow diagram; participant flow throughout trial; reasons for non-participation/withdrawal; target sample sizes.

**Results:**

133 reports were reviewed. Overall, 79% included a flow diagram, but over a third were incomplete. The majority reported the flow of participants at each stage of the trial after randomisation. However, 40% failed to report the numbers assessed for eligibility. Percentages of participants retained at each stage were high: for example, 90% of eligible individuals were randomised, and 93% of those randomised were outcome assessed. On average, trials met their sample size targets. However, there were some substantial shortfalls: for example 21% of trials reporting a sample size calculation failed to achieve adequate numbers at randomisation, and 48% at outcome assessment. Reporting of losses to follow up was variable and difficult to interpret.

**Conclusion:**

The majority of RCTs reported the flow of participants well after randomisation, although only two-thirds included a complete flow chart and there was great variability over the definition of "lost to follow up". Reporting of participant eligibility was poor, making assessments of recruitment practice and external validity difficult. Reporting of participant flow throughout RCTs could be improved by small changes to the CONSORT chart.

## Background

Randomised controlled trials (RCTs) are widely acknowledged as the design of choice for evaluating the effectiveness of health care interventions. However, poor recruitment and retention of eligible participants is common and problematic because the sample may fail to be representative of the relevant population [1], the comparability between treatment arms may be lost, and statistical power may be reduced [2]. For example, a review that considered the evidence on participation in RCTs suggests that treatment effects may be exaggerated by low participation due to inclusion of participants with a greater capacity to benefit [3]. In addition to being part of the good design and conduct of trials, clear and sufficiently detailed reporting of participant flow is important in order for readers to assess the generalisability [4], relevance and comparability of trial results.

The CONSORT (*Con*solidated *S*tandards *o*f *R*eporting *T*rials) statement, which consists of a checklist of items to be addressed, was developed to improve poor reporting [5-9]. CONSORT specifically addresses the participant flow through RCTs, providing a template flow diagram for authors. First published in 1996, the CONSORT statement was subsequently revised to provide greater clarity and to extend the flow diagram [10,6]. CONSORT is now supported by organisations including the International Committee of Medical Journal Editors, the World Association of Medical Editors, and the Council of Science Editors [5]. There is growing evidence that CONSORT has improved the quality of RCT reports [5,6,8,9], although previous reviews also suggest that problems remain. For example, Gross et al. (page 3 [7]) found "sporadic and incomplete reporting of the recruitment process in many RCTs"; Devereaux et al. (page 384 [5]) found "suboptimal reporting" of 11 key methodological factors even among journals supporting CONSORT; Folkes et al. (page 845 [11]) found that "information pertaining to pre-randomization was often missing or incomplete"; and Mills et al. (page 485 [12]) found that "even after a revision of the CONSORT statement and subscription to it by leading journals, the reporting of key methodological items continues to be poor".

Previous reviews have focused on: specific aspects of participation (such as recruitment or pre-randomization) rather than the process as a whole [7,11]; reporting of different stages of participation without assessing (or with only limited assessment of) the flow of participants through the trial [5,6,9]; and almost exclusively on trial reports published prior to the revised CONSORT statement and flow diagram in 2001. Evaluating reports published in 2004 (i.e. well after the publication of the revised CONSORT statement), the present review updates these findings. It aims to assess the adequacy of reporting of participant flow through RCTs (from the point of eligibility assessment to the primary analysis), and the levels of such participation, in general and in comparison with sample size targets. By focusing on high quality journals with wide readerships, this review aims to assess the state of the art in recruitment, retention and reporting of those activities.

## Methods

### Literature search

We searched Medline for all reports of RCTs published between July and December 2004 in six major journals: *Annals of Internal Medicine*, *Annals of Surgery*, *British Medical Journal (BMJ)*, *The Journal of the American Medical Association (JAMA)*, *The Lancet*, and *The New England Journal of Medicine (NEJM)*. These journals were selected for their high quality and influence. They were also chosen to span both medicine and surgery, capturing the variation in recruitment and retention challenges facing different fields of enquiry, and reflecting our particular interest in trials of surgery. We used the standard RCT filter [13], limited to trials involving human participants. One reviewer screened all titles and abstracts for relevance and a second independently crosschecked a sample of 50%. Discrepancies were resolved through discussion of the full paper.

### Inclusion/Exclusion criteria

**Figure 1:**
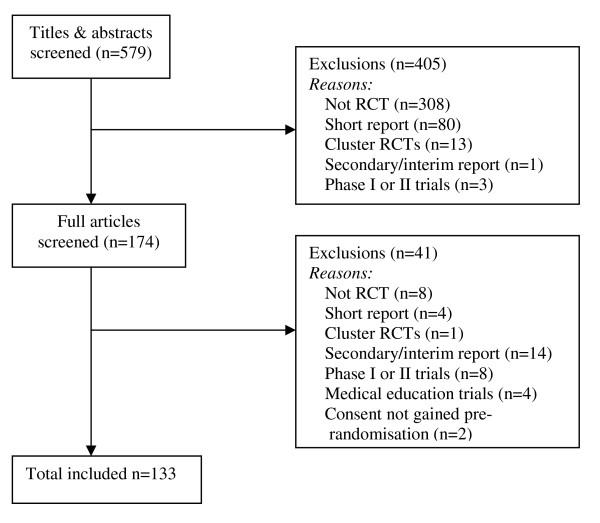
**Flow of papers through the systematic review**.

We included all Phase III RCTs of interventions designed to improve health outcomes and/or processes, in which randomisation was at the individual level. Phase III trials were defined as those conducted in a clinical setting, with patients representative of normal referrals to that setting, and with the aim of estimating intervention effectiveness. Cluster and medical education trials were excluded, as were short, secondary or interim reports, and Phase I and II trials. Compared to Phase III trials, Phase 1 and II trials make different demands with regards to recruitment and retention, typically requiring smaller sample sizes, including a larger number of treatment arms varying in dosage, using shorter term outcomes, making greater use of sequential testing methods, and aiming to establish the treatment effect in ideal conditions. Although we did not exclude studies where the report failed to state explicitly that it was Phase III, we did exclude those that were explicitly labelled Phase I/II. Where there was any lack of clarity, at least one other team member was consulted by the reviewers. In total, eleven Phase II trials were excluded (Figure 1), primarily because they were concerned with efficacy or dose finding.

### Data extraction

For each paper, two independent reviewers extracted data using a standardised form (papers were divided between three reviewers: MT, IdS and ZT. Each paper was read by two of the three reviewers). Disagreements were resolved by consensus in discussion with a third party (depending on the nature of the disagreement, one or more of the following authors was consulted on the basis of their area of expertise: SB, CM, JD and TP). For each included trial, the following information was recorded: general trial details (number and nature of interventions, number of centres); inclusion of a complete or partially complete revised CONSORT flow diagram; data on participant flow throughout the trial (extracted from text, tables and diagrams); reasons for non-participation and withdrawal; and data on target sample sizes according to *a priori *power calculations.

### Analysis

There are four key stages of an RCT: eligibility assessment, randomisation, treatment and outcome assessment. Since data were often skewed, percentages progressing from one stage to the next were reported as median rates (with inter-quartile ranges – IQR). Each study made an equal contribution to these statistics, irrespective of the overall sample size. To allow comparability across studies, we considered rates of outcome reporting at the first post-treatment assessment. For trials including details of an *a priori *sample size calculation, median rates of participation were also calculated at each stage of the trial in relation to the sample size required to provide an adequately powered analysis. Associations between trial characteristics and levels of participation were investigated using Mann-Whitney (for characteristics with two levels), Kruskal-Wallis (three or more unordered levels) or trend tests (three or more ordered levels) [14]. All statistical analyses were conducted using Stata 9 statistical software (StataCorp, College Station, Tx, 2005).

## Results

### Profile of RCT reports

The search identified 579 potential papers. A total of 133 RCT reports were included in this analysis, with most exclusions due to the absence of randomisation or because the paper was not a full-length report (Figure 1). Of the 133 included trials, nearly one-third were published in the *NEJM*, a quarter in *The Lancet *and around a fifth in *JAMA *(Table 1). The majority were two-armed, multi-centre, parallel group trials, with external funding. Just over half assessed drug interventions, and there were similar numbers with active and placebo control arms. The time from randomisation to the first post-treatment assessment was obtainable for 129 studies. Of these, around 20% lasted up to four weeks, a quarter lasted more than four weeks, just over a quarter lasted more than six months, and a further quarter lasted more than eighteen months. Two of the missing studies failed to report this information, and two had this information defined by the outcome (in hospital death and the end of a variable length counselling programme) and did not provide further details. This variable is a little inconsistent in its definition. Some studies have a defined follow-up appointment, making it easy to derive this information from the report. Others follow individuals until they experience an event (e.g. death) with median follow-up most commonly reported in those cases.

**Table 1:** Characteristics of the 133 included RCT reports

**Trial characteristic**		**Number of Included RCT Reports (%)**
*Journal*	NEJM	39 (29.3)
	Lancet	31 (23.3)
	JAMA	25 (18.8)
	BMJ	15 (11.3)
	Annals of Internal Medicine	13 (9.8)
	Annals of Surgery	10 (7.5)

*Sample Size*	<200	33 (24.8)
	200–449	37 (27.8)
	450–749	31 (23.3)
	750+	32 (24.1)

*Centres*	Multiple	95 (71.4)
	Single	38 (28.6)

*Study Design*	Parallel	125 (94.0)
	Factorial	5 (3.8)
	Crossover	3 (2.3)

*Number of Arms*	Two	103 (77.4)
	Three	14 (10.5)
	Four	12 (9.0)
	Five	2 (1.5)
	Six	2 (1.5)

*Interventions*	Drug	68 (51.1)
	Surgery	21 (15.8)
	Allied/Complementary Medicine	20 (15.0)
	Other^a^	24 (18.1)

*Nature of Control Arm*	Active Control	63 (47.4)
	Placebo Control	70 (52.6)

*Funding Source*	External^b^	123 (92.5)
	Internal/Not stated^c^	10 (7.5)

^a ^For example, care management, patient education, radiotherapy or mixed interventions

^b ^For example, pharmaceutical companies, government agencies, charities etc.

^c ^For example, hospital department where trial took place; no additional external funding obtained

### CONSORT flow diagram

Overall, nearly 50% (64/133) included a complete revised CONSORT flow diagram, and 31% (41/133) a partial diagram (Table 2). All trials reported in *JAMA*, *BMJ *and *The Lancet*, and all but one in *Annals of Internal Medicine*, included at least a partial CONSORT diagram. However, within *Annals of Surgery *and *NEJM *at least half of trials failed to report any form of CONSORT flow diagram.

**Table 2:** Trial reports providing a revised CONSORT diagram

Journal	Yes completely (%)	Yes partially (%)	No (%)
JAMA (n = 25)	20 (80.0)	5 (20.0)	0

Annals Int. Med. (n = 13)	10 (76.9)	2 (15.4)	1 (7.7)

BMJ (n = 15)	10 (66.7)	5 (33.3)	0

Lancet (n = 31)	16 (51.6)	15 (48.4)	0

NEJM (n = 39)	7 (17.9)	10 (25.6)	22 (56.4)

Annals Surgery (n = 10)	1 (10.0)	4 (40.0)	5 (50.0)

Total (n = 133)	64 (48.1)	41 (30.8)	28 (21.1)

**Figure 2:**
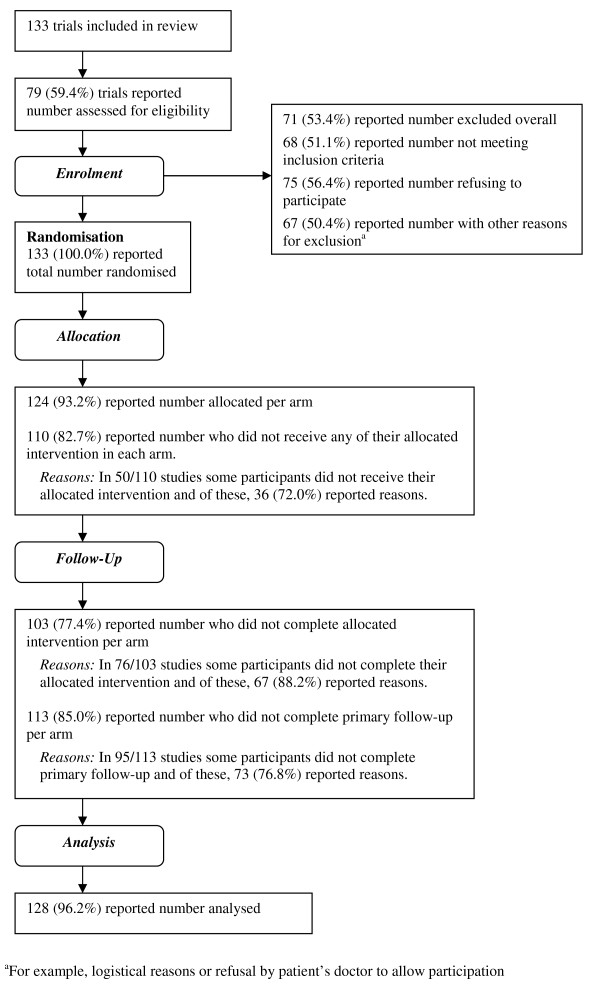
**CONSORT diagram of reporting of participant flow**.

Figure 2 presents a revised CONSORT diagram of the levels of reporting for the whole process of participant flow. Reporting was far better after the point of randomisation than before. Trials providing only partial CONSORT diagrams most commonly omitted data from the eligibility and recruitment phases of the trial. Only just over half the trials reported the numbers excluded during these phases. Reasons for exclusions were poorly reported with only around 50% reporting the number of individuals not meeting the inclusion criteria and 50% reporting the number refusing to participate. All trials reported the total number randomised, the majority (93%) the number allocated per arm, 77% the numbers of participants completing treatment, 85% completing follow-up, and 96% the numbers analysed. Where participants did not complete allocated treatment or primary follow-up, reasons were given in 88% and 77% of cases respectively.

### Recruitment, randomisation and retention rates

Table 3 shows the median rates of progression through each of the key stages of a trial. Since reporting was variable (Figure 2), the number of trials contributing data at each stage varies. The percentages of individuals retained at each stage of the trials were, in general, high. The biggest hurdle to full participation was at the stage of initial assessment for eligibility, with an average of 30% of invitees failing to attend and 30% of those attending found to be ineligible. Of individuals assessed as eligible, the median percentage randomly allocated to a treatment arm was 90%, with a quarter of studies reporting 100% being randomised. Nevertheless, the range was wide, with a further quarter of studies randomising 77% of eligible individuals or fewer and the poorest performing trial randomising only 20%. Some 28% of studies (33 of 120 trials providing information) found that at least one patient did not meet the eligibility criteria after they were randomised. Whilst on average this percentage was minimal, with 90% of trials reporting less than 2% failing to meet the eligibility criteria post-randomisation, around 6% reported rates over 5% and one trial found 25% of participants to be ineligible after randomisation.

**Table 3:** Reported rates of participant flow through the trial (reported as median rates across studies)

Current stage^a^	Next stage^a^	Number of studies^b^	Median progressing to next stage	First and third quartiles	Minimum and maximum
Invited to eligibility assessment	Attended eligibility assessment	13	70%	44%, 96%	15%, 100%

Attended eligibility assessment	Found to be eligible	71	70%	49%, 88%	6%, 100%

Found to be eligible	Randomised to a treatment arm	75	90%	77%, 100%	20%, 100%

Randomised to a treatment arm	Outcome assessed	113	93%	86%, 99%	49%, 100%

Outcome assessed	Included in analysis	111	100%	100%, 103%^c^	100%, 202%^c^

^a ^'Current stage' denotes the denominator and 'Next stage' the numerator for the percentage progressing to next stage within each study

^b ^Relates to the number of studies providing relevant data

^c ^In some instances missing outcome data were imputed, hence percentages greater than 100% are possible here

Seventy studies specifically reported the proportion of eligible individuals *refusing *to be randomly allocated to treatment; whilst the median rate was 5% (IQR 0%–12%), one study reported as many as 61% refusing randomisation. In general, trials did well at retaining participants from the point of randomisation through to outcome assessment. Losses were reported at different points throughout the trials. Of those randomised a median of 0% (IQR 0% to 2%) were lost before having received any of the allocated treatment and 7% (IQR 0% to 15%) after having received some, but not all, of the allocated treatment. Again, there were instances where specific studies performed very poorly in these respects. For example, in one study 34% of participants were lost without receiving any of the allocated intervention and in another, 50% were lost having received only part of the treatment. Of those randomised, a median of 93% (IQR 86% to 99%) provided an assessment of outcome (Table 3). Outcome data were often imputed (using different methods such as last observation carried forward) where data were missing, which accounts for the fact that in a quarter of the trials, more participants were included in the analysis than had their outcome assessed (Table 3). Where outcome data were available they were always included in the analysis. There was considerable difficulty in extracting data about losses to follow-up because of different interpretations of what was meant by "lost to follow-up".

### Extent to which trials met sample size targets

Most of the trials (112/133; 84%) reported a sample size calculation. The power and significance levels used in these calculations varied across trials, although all were reported to be 80% or greater and 5% or less respectively. In addition, some trials inflated the required sample size in order to allow for potential dropouts. Where this was reported, the target sample size (that is, the uninflated estimate) was recorded and used in the percentages below. Relative to the target sample size, the majority of trials either met or exceeded their goal across all stages of recruitment and retention (Table 4). Trials initially assessed a median of 230% of their target number, identified as eligible a median of 130%, and randomised a median of 110% of the required sample size. Trials also tended to meet their sample size targets right through to the point of outcome assessment (median = 100%), with three quarters of the trials assessing at least 92% of the target number of participants at outcome. Whilst these average figures are high, there remain a number of trials failing to achieve the necessary sample size. For example, 21% of trials reporting a sample size calculation failed to achieve adequate numbers at randomisation, and 48% at outcome assessment. Moreover, the worst performing trials in this respect fell substantially under target with one trial randomising only 43% of the required number and another assessing only 36% at outcome.

**Table 4:** Reported rates of participant flow through the trial as a percentage of the number required by the reported sample size calculation

	Number of studies^a^	Median % of sample size required	First and third quartiles	Minimum and maximum
Invited to screening	12	410%	288%, 951%	131%, 2549%

Attend screening	62	230%	132%, 379%	83%, 2361%

Eligible	58	130%	108%, 160%	72%, 431%

Randomised	106	110%	100%, 128%	43%, 213%

Outcome assessed	94	100%	92%, 111%	36%, 158%

In analysis	102	103%	98%, 117%	43%, 169%

7 studies which stopped early having demonstrated a benefit of treatment are excluded from these analyses

^a ^Relates to the number of studies providing relevant data

Note: the α (significance) and 1-β (power) levels used in each trial's sample size calculation may vary

### Potential explanatory variables

Table 5 presents the results of an exploratory analysis investigating associations between recruitment and retention rates and trial characteristics. While there is evidence of an association between study size and the proportion of people screened who were found to be eligible to participate (p = 0.026), there is no simple pattern to the relationship. Two arm trials were found to assess outcome in a higher proportion of those randomised when compared to trials with three or more arms (p = 0.0035). Also, surgery trials were found to assess outcome in a higher proportion of those randomised when compared to studies of other treatment approaches (p = 0.014). Studies funded by government or charities randomised a lower proportion of those screened as eligible compared to studies receiving industry funding or which were internally funded (p = 0.048). There was no convincing evidence of differences in recruitment and retention between multi-centre and single-centre studies, or between studies with active and placebo controls.

**Table 5:** Associations between recruitment and retention rates and trial characteristics

	Screened and eligible			Eligible and randomised			Randomised and outcome assessed		
	
Factor & level	Median	IQR	Number of studies	Median	IQR	Number of studies	Median	IQR	Number of studies
Study size									
<200	78	63, 95	25	90	79, 100	26	92	86, 94	32
200–449	71	56, 95	18	93	78, 99	21	92	86, 99	29
450–749	51	33, 64	15	86	66, 97	15	93	84, 98	23
750+	71	41, 80	13	91	85, 100	13	97	89, 100	29
			*p = 0.026*			*p = 0.99*			*p = 0.24*
*Number of arms*									
2	72	48, 93	56	90	78, 100	59	94	88, 99	89
3+	68	51, 79	15	89	70, 97	16	86	76, 94	24
			*p = 0.33*			*p = 0.75*			*p = 0.0035*
*Multi-centre?*									
Yes	75	42, 86	44	92	73, 99	47	94	85, 99	79
No	64	50, 94	27	87	78, 100	28	93	88, 96	34
			*p = 0.91*			*p = 0.70*			*p = 0.64*
*Treatment focus*									
Drug	71	53, 86	29	94	73, 100	30	94	86, 99	56
Surgery	75	48, 99	10	89	63, 100	10	99	92, 100	17
Allied	67	50, 88	17	91	81, 99	18	90	85, 94	18
Other	64	44, 86	15	86	79, 93	17	92	84, 96	22
			*p = 0.89*			*p = 0.56*			*p = 0.014*
*Control*									
Active	70	51, 86	30	88	74, 95	32	92	85, 98	52
Placebo	68	49, 88	41	94	77, 100	43	93	86, 99	61
			*p = 0.82*			*p = 0.24*			*p = 0.46*
*Time to assessment*									
0 to 4 weeks	8	33, 86	*10*	83	63, 94	*11*	99	93, 100	*26*
>4 weeks to 6 months	7	51, 78	*23*	91	81, 100	*24*	92	87, 94	*30*
>6 to 18 months	74	40, 93	*20*	94	72, 99	*21*	88	79, 98	*31*
>18 months	75	63, 93	*16*	91	83, 100	*16*	95	86, 100	*23*
			*p = 0.087*			*p = 0.242*			*p = 0.075*
*Funding*									
Pharma	74	54, 93	*28*	94	83, 100	*29*	93	85, 99	*56*
Government/charity	67	48, 86	*38*	85	70, 97	*40*	93	86, 96	*49*
Internal/unstated	56	51, 56	*5*	98	94, 100	*6*	95	91, 100	*8*
			*p = 0.51*			*p = 0.048*			*P = 0.50*

P values were obtained from Mann-Whitney tests (for characteristics with two levels), Kruskal-Wallis tests (three or more unordered levels) or trend tests (three or more ordered levels)

## Discussion

The majority of 133 trials published in six high quality journals during 2004 reported the flow of participants at each stage of the trial after the point of randomisation (over 75% for each stage). The percentages of individuals retained at each stage of the trials were high, with the median percentage of those eligible randomised, those randomised assessed at outcome, and those assessed included in the analysis, all being above 90%. On average, 100% of trial participants received at least part of their allocated intervention and 93% received it all. However, percentages of patients reported up to the point of randomisation were less impressive, with 40% failing to report the numbers assessed for eligibility, and similar numbers failing adequately to report reasons for exclusions. A small proportion of trial reports had very poor rates of reporting and/or recruitment and retention, despite the fact that they were published in high quality journals.

Overall, this review supports the growing body of evidence that the CONSORT statement has improved reporting of RCTs [5,6,8,9]. Comparison of the present sample with a similar review of trial reports published in 1998 [6] shows numbers of reports including at least a partial diagram increasing from around 50% in the previous review (139 of 279 reports) to almost 80% here (105 of 133 reports). The *NEJM *continues to contribute a substantial proportion without any flow diagram (44%), although this percentage was lower than previously (92%) [6]. The *NEJM *was the last of the five journals to accept the CONSORT statement officially, in January 2004. It is likely, therefore, that some papers accepted for publication in the *NEJM *prior to adoption of the CONSORT statement were included in this review. It is also possible that some reports omitted a CONSORT flow diagram in accordance with the CONSORT guidelines, which state that it may be unnecessary to include a diagram for simple trials with no losses to follow up or exclusions. However, it seems likely that this would only explain, at most, a very small minority of omissions for two reasons: first, for most trials published in these journals it is very unlikely that there will have been no losses to follow up or exclusions; and second, as Table 2 suggests, the inclusion of the diagram seems to depend much more on whether the journal requires compliance with the whole CONSORT package.

This review demonstrates that whilst the percentage of papers providing adequate data to complete each element of the CONSORT flow diagram is higher than pre-CONSORT [7,8], there is evidence of continued poor reporting of pre-randomisation figures [11]. This may partly reflect greater difficulty in recording data when patients are not yet part of a trial, and also the view that these are less important than figures from randomisation onwards. However, from a practising clinician's viewpoint, these figures are crucial for judging whether the results of a trial are generalisable to the patients s/he may treat [1]. If only a proportion of those patients who fulfil the clinical eligibility criteria are included in the trial, then there is scope for trial participants to be unrepresentative of the population of eligible patients as a whole. This may allow important differences to arise between the trial participants and patients seen in clinical practice who fit the clinical description of trial participants.

In terms of percentages of participants retained at each stage of an individual trial, we found that, on average, only 70% of those invited to screening attended. Only 13 studies reported these data, but this suggests that many potentially eligible patients exclude themselves for reasons unknown to the investigators. Potential reasons may include refusal to attend the initial assessment, to be randomised, or to be involved in research altogether, or may be related to logistical reasons. In turn, this means that the true rate of refusal to be randomised (calculated in this review from the number of eligible patients refusing randomisation and estimated to be on average 5%) may be higher than observed here and the representativeness of the final samples may be reduced.

On average 70% of those screened were found to be eligible, although it was often not clear how many potential eligible patients did not attend for screening. If eligibility criteria are appropriate and correctly applied, even very low eligibility rates do not necessarily indicate poor trial practice as this will depend on the condition under study. However, large numbers of exclusions can indicate that eligibility criteria are too narrow, threatening the generalisability of the sample. Some reports conflated those not eligible with participants attending screening who then declined participation, using informed consent as an eligibility criterion, making it impossible to calculate separate eligibility and refusal rates [7]. This lack of clarity is likely to have contributed to a quarter of trials reporting 100% of eligible individuals being randomised. Such rates might be plausible for simple placebo-controlled trials, but in pragmatic trials the possibility of coercion or inadequate information cannot be ruled out.

A strength of this review is that it has used an additional measure for assessing participation: comparing achieved participation rates with targets set by prior sample size calculations, although it was not clear whether this was as originally reported in the protocol. Overall, 84% of trials reported a sample size calculation – a finding that echoes a previous review of reports published in 2002 ([12], but see [15] for evidence that some of the required parameters of the calculations are often not reported). On average, trials met their sample size targets at all stages of the trial, including (most crucially) at outcome assessment and analysis. This was generally achieved by over-recruiting participants, especially for eligibility assessment. A median of more than double the target sample size was assessed for eligibility, with a quarter of trials assessing almost four times the target. However, 48% of trials did not reach their targets, albeit only marginally for many (a quarter achieving 92% or more at outcome assessment). In general, such shortfalls relate to difficulties in recruiting study participants rather than losses post-randomisation.

The recruitment and subsequent retention of participants through a trial will be heavily dependent on the nature of the study and its interventions as well as the organisation of the trial. This review found that trials with more than two arms were slightly worse at retaining participants to the point of outcome assessment than those with only two arms, that surgical trials were better at retaining participants than other trials, and that government- or charity-funded studies randomised a lower proportion of those screened as eligible than studies funded by industry or internally. A previous meta-analysis [16] of acute stroke trials found that, after adjusting for the stringency of the eligibility criteria, trials with a large number of study centres tended to recruit less efficiently than those with fewer centres.

The degree of retention seen in this review could be an over-estimate, since poorly recruiting or retaining trials will be less likely to be published in such high quality journals. A recent review [17] of registered (rather than published) trials found that more than two thirds failed to reach their recruitment target, although a further quarter achieved over 80%. Other reviews [18,19] of registered trials have demonstrated a similar percentage of trials failing to reach targets, but with a wider range of deficiency among those failing. The most common reason cited was recruitment difficulties.

There are several limitations to this study. The findings reflect reporting in the six journals under review, leaving open the question of the extent to which this reflects reporting of RCTs in general. While our focus on the six journals limits the generalisability of our findings, it should not limit the applicability of our recommendations. Fuller reporting of pre-randomisation patient flow will be as informative in specialist journals as in the high quality general medical journals we reviewed. A further limitation was the focus on papers published in 2004. This date was chosen as RCTs from this time period were likely to be listed on Medline when we started work on this review and it was not possible to up-date it. As the CONSORT recommendations for reporting patient flow in trials have not changed since 2004, we believe that our findings are likely to be a true reflection of current reporting in high quality journals. There is also the question of whether the reporting in the journals really reflects what happened in the course of these RCTs. There is evidence, for example, that some important methodological steps may be undertaken in trials but not reported [20]. It is also possible that trials with poorer participation may be less likely to report figures for participant flow. Hence our recruitment and retention rates may be over-estimates. We made no attempt to trace any missing information or data from RCTs, since the aim was to assess the reporting of trials, but this would be an important aim for future research.

**Figure 3:**
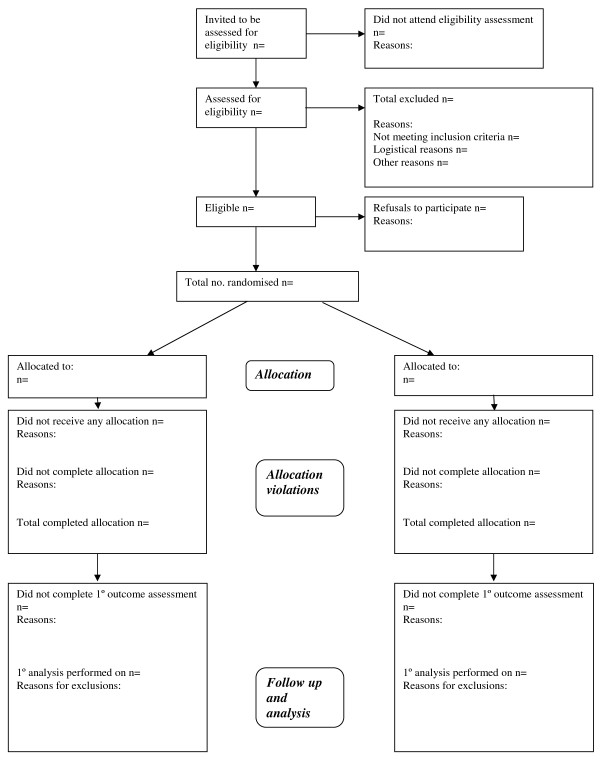
**Proposed revised CONSORT flow diagram**.

With respect to the CONSORT guidelines, there are recommendations that can be made on the basis of the findings of this review. We would propose that additional boxes be included in the flow diagram for the number invited to attend initial assessment for eligibility and the number not attending following such an invitation, where this is possible (see Figure 3). Ideally, the latter should also include a list of reasons so that refusals to be randomised that occur at this point can be determined. We would also suggest that informed consent be kept separate from the eligibility criteria. We recognise that it will not always be possible to provide all these figures even when a good data management system is in place. For example, where potential participants are invited to an eligibility assessment through posters or newspaper adverts, it will not be possible to provide a figure for the total number who read the advert. However, their inclusion should be advocated wherever possible to enable better assessment of generalisability and estimation of a true refusal rate for an individual trial. They would also be useful for management of recruitment during trial operation.

The CONSORT flow diagram contains a single box labelled "follow-up" in which authors are required to state the number "lost to follow-up" and "discontinued intervention" (with reasons). The definition of "lost to follow up" varied considerably in these papers. Our interpretation of "lost to follow-up" is any participant who is not assessed on the primary outcome irrespective of whether or not they complete their allocated intervention, but these two events can happen in a number of combinations (e.g. a participant might fail to complete their allocation and their outcome assessment or might complete their allocation but fail to be outcome assessed etc.). These two terms are not used consistently by authors, making it difficult to be certain at what point in the trial a participant was lost. "Lost to follow-up" could be replaced with "number not completing primary outcome assessment" (Figure 3). Details of those who "discontinued intervention" may serve better as a separate box related to allocation violations, which would precede outcome assessment in the natural flow of participants through a trial. It could be reported in the text, but since such information is important in assessing to what extent the estimated effectiveness of the intervention might be under- or overestimated, it is useful to have in the CONSORT diagram. Greater clarity over the definition of "lost to follow up" would be helpful.

Finally, there is currently no place within the CONSORT diagram for those found to be ineligible after randomisation. Such individuals are sometimes classed as "lost to follow-up" and excluded from the analysis. Although usually a small number, it is perhaps clearer to report such ineligibles in the text of a trial report or as a footnote to the CONSORT diagram, but to fully report reasons for exclusions from the primary analysis in the diagram.

## Conclusion

This review provides evidence of good reporting of participant flow after randomisation in RCTs published in six major journals. However, reporting of participant eligibility was poor, with only two-thirds of the reports including a complete CONSORT flow chart, and few RCTs providing sufficient information, even where it would be easy to do so, to allow judgement of the trial's generalisability to patients in clinical practice. In addition, there was great variability in the definition of "lost to follow-up" and consequently its impact on data analysis. Assessing the adequacy of participation in trials is an important component of monitoring the quality of trial results because of the impact that recruitment and retention difficulties can have on a trial's representativeness and the comparability of its treatment arms. Even in high quality journals there remain trials with very poor recruitment and retention rates and poor reporting. The inclusion of a small number of additional data items relating to eligibility and loss to follow-up would further improve the CONSORT flow chart and enable better evaluation of recruitment and retention in published trials.

## Competing interests

The authors declare that they have no competing interests.

## Authors' contributions

JD, TP, JS were responsible for the study concept and design. MT, IdS, ZT, SB, CM, JD, TP carried out the data extraction. SB, CM, MT performed the analysis and interpretation of data. MT and SB produced the first draft of the manuscript. SB, CM, JD and TP provided critical revisions of the manuscript. All authors read and approved the final manuscript. JD is the guarantor of the manuscript.
